# Intrascrotal Dedifferentiated Leiomyosarcoma Originating from Dartos Muscle

**DOI:** 10.1155/2014/841929

**Published:** 2014-12-11

**Authors:** Taro Teshima, Sayuri Takahashi, Shoichi Nagamoto, Hideyo Miyazaki, Tohru Nakagawa, Tetsuya Fujimura, Hiroshi Fukuhara, Haruki Kume, Yukio Homma

**Affiliations:** Department of Urology, The University of Tokyo, 7-3-1 Hongo, Bunkyo-ku, Tokyo 113-8655, Japan

## Abstract

A 46-year-old man, who had visited our hospital complaining of a small intrascrotal nodule ten years ago, returned to us because of the rapid growth of the nodule. Computed tomography revealed a heterogeneously enhanced intrascrotal tumor of approximately 4 × 3 cm. The tumor and the right testis were excised with the adhered right scrotal skin. The pathological diagnosis was pleomorphic leiomyosarcoma with dedifferentiation originating from the dartos muscle. Urological dedifferentiated leiomyosarcomas are rarely reported and the clinical features are mostly unknown. This is the first report to describe the dedifferentiated leiomyosarcoma of the dartos muscle.

## 1. Introduction

Dedifferentiation is a well-recognized process in several bone and soft tissue tumors, including liposarcoma, chondrosarcoma, periosteal osteosarcoma, chordoma, and solitary fibrous tumor [1]. However, dedifferentiation of leiomyosarcomas is very rare. Herein we report a case of dedifferentiated leiomyosarcoma originating from dartos muscle.

## 2. Case Presentation

**Figure 1:**
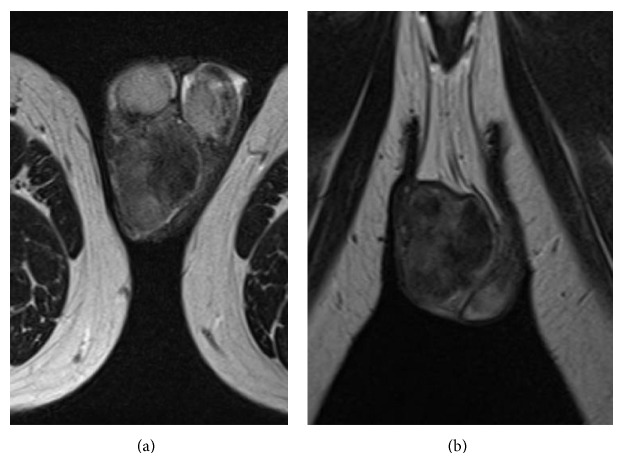
MRI T2 transverse (a) and coronal (b) images show a mass in the right paratesticular region.

**Figure 2:**
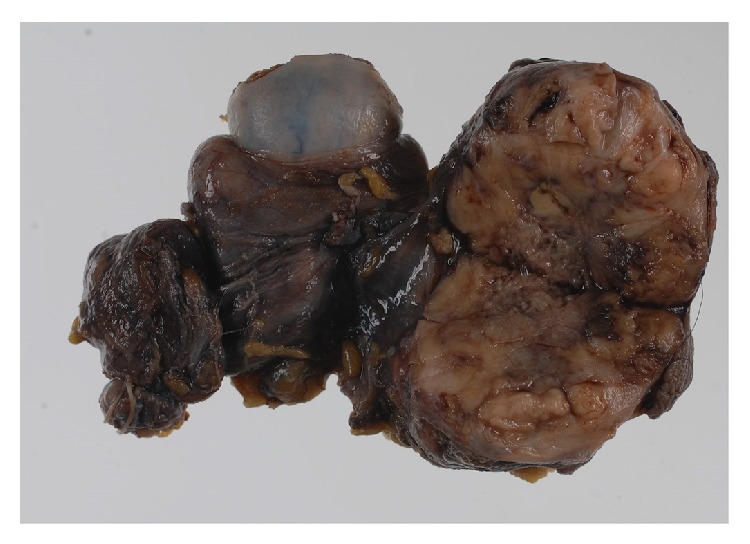
Macroscopic finding. The mass is separated from the testis and epididymis.

In 2004, a 46-year-old man visited our hospital with a complaint of a small intrascrotal nodule and was placed under observation. In January 2014, he noticed rapid growth of the nodule and visited us again. Physical examination showed a mass in the scrotum near the right testis. Blood tests yielded no specific results. Contrast-enhanced computed tomography revealed a heterogeneously enhanced tumor of approximately 4 × 3 cm. Magnetic resonance imagingshowed a heterogeneous signal in T2 weighted image and early contrast enhancement and washout (Figure 1). There was no evidence of metastasis. Under clinical diagnosis of a malignant intrascrotal tumor, we excised the scrotal tumor with the adhered skin and the right testis. The tumor was yellow in color and 4.7 cm in the maximum diameter. It was located beneath the scrotal skin, apart from the spermatic cord or testis (Figure 2).

**Figure 3:**
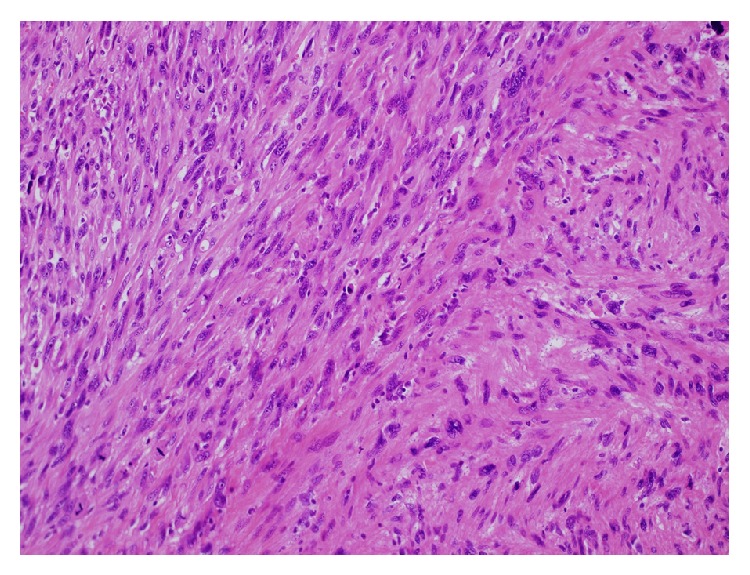
Microscopic finding. Spindle shaped leiomyosarcoma cells (right side) and what appeared to be MFH with a high mitotic rate (left side). Hematoxylin and eosin stain ×20 (upper).

**Figure 4:**
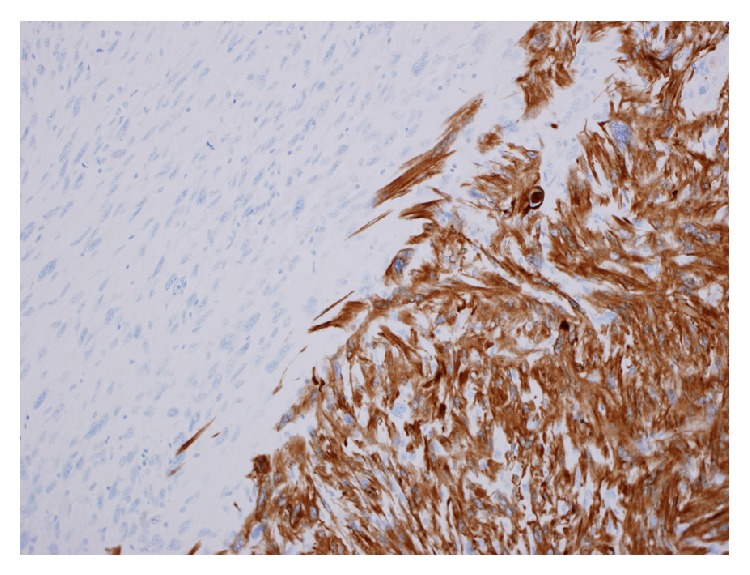
Leiomyosarcoma (right) is stained by antibody against calponin but dedifferentiated leiomyosarcoma (left) is not. ×20 (lower).

Microscopically, the tumor consisted of two different components: leiomyosarcoma and malignant fibrous histiocytoma-like dedifferentiated sarcoma (Figure 3). Immunohistochemistry detected dedifferentiated components of leiomyosarcoma, which were characterized by lack of staining with muscle markers except for caldesmon (Figure 4, Table 1). The pathological diagnosis was pleomorphic leiomyosarcoma with dedifferentiation originating from the dartos muscle of the right scrotum. Immunohistochemical stains for MDM2 and CDK4 were negative; therefore, we excluded dedifferentiated liposarcoma with myogenic differentiation. Although the surgical margin of specimen was negative, there were multiple tumor invasions to peripheral veins. The dermis was invaded, but the epidermis was intact.

The patient had no evidence of recurrence at six months after the operation.

## 3. Discussion

Soft tissue sarcomas are a heterogeneous group of nonosseous tumors that arise from the embryonic mesoderm [2]. In this group of tumors, genitourinary (GU) sarcoma is relatively rare. It is estimated that approximately 10,000 new patients are yearly diagnosed with soft tissue sarcomas in the USA [3], of which GU tract sarcomas consist of 2.1% only [4]. Dotan et al. reported that among 131 GU tract sarcomas the most common histological type was leiomyosarcoma (19%) [2]. Coleman et al. reported that the spermatic cord was the most common site of GU sarcomas (30%) [5]. Of 24 cases of leiomyosarcoma of paratesticular region, only one case was reported to have the origin in the dartos muscle [6]. Our case is the first report of dedifferentiated leiomyosarcoma originating from the dartos muscle.

Chen et al. reported that dedifferentiated leiomyosarcoma was seen in 1.4% of all leiomyosarcomas consulted from 1991 to 2007 [1]. In their report, these dedifferentiated leiomyosarcomas lacked the characteristic immunohistochemical staining of differentiated leiomyosarcoma for muscle-specific actin, smooth muscle actin, desmin, and CD34 [1]. Our case showed the similar features; that is, the differentiated component showed to be strongly positive for muscle markers, but the dedifferentiated component was negative (Table 1).

As for grading systems of sarcomas, the French Federation of Cancer Centers Sarcoma Group grading system has been shown to be reproducible among pathologists and correlate with the clinical outcome [7, 8]. In this French system, mitotic activity and the amount of tumor necrosis are scored individually, and these scores are summed up to give a final score of the sarcoma grade [7]. Following this grading system, the score of differentiation of our case was three, the score of necrosis one, and the score of mitotic activity three. The total score was seven, and our case was rated as grade 3.

The optimum local and systemic treatment for these tumors remains controversial, but there is a general consensus that all paratesticular sarcomas in adults should be managed with complete resection, including high ligation of the spermatic cord [7]. Prognosis of GU sarcomas depends on tumor size, grade, stage, histologic type, and lymph node involvement [9–13]. Froehner et al. indicated the tumor size over 5 cm as an important prognostic factor [14]. Of 14 patients of paratesticular leiomyosarcoma, four (29%) had local recurrences and one had metastases [6]. Galosi et al. recommended adjuvant radiation after radical surgery for the high rate of local recurrences [15]. Chen et al. reported a worse prognosis of dedifferentiated sarcomas compared with differentiated sarcomas; of 13 dedifferentiated leiomyosarcomas, metastasis occurred in five (38%) and local recurrence in five (38%) [1]. Close follow-up is needed because of a high frequency of recurrence and metastasis of dedifferentiated leiomyosarcoma.

## Figures and Tables

**Table 1:** Immunostaining of our case.

	Smooth muscle sarcomatoid	Malignant fibrous histiocytoma-like tumor
Vimentin	++	++
*α*-Smooth muscle actin	++	−
Desmin	++	−
Muscle-specific actin	++	−
Calponin	++	−
Caldesmon	++	+
CD99	Focal+	+
CDK4	−	−
MDM2	−	−
S100	−	−
CD34	−	−
AE1/AE3	−	−
CAM5.2	−	−
MIB-1 index	5%	50%

## References

[1] Chen E., O'Connell F., Fletcher C. D. M. (2011). Dedifferentiated leiomyosarcoma: clinicopathological analysis of 18 cases. *Histopathology*.

[2] Dotan Z. A., Tal R., Golijanin D., Snyder M. E., Antonescu C., Brennan M. F., Russo P. (2006). Adult genitourinary sarcoma: the 25-year memorial sloan-kettering experience. *Journal of Urology*.

[3] Jemal A., Murray T., Ward E. (2005). Cancer statistics, 2005. *CA: A Cancer Journal for Clinicians*.

[4] Stojadinovic A., Leung D. H. Y., Allen P., Lewis J. J., Jaques D. P., Brennan M. F. (2002). Primary adult soft tissue sarcoma: time-dependent influence of prognostic variables. *Journal of Clinical Oncology*.

[5] Coleman J., Brennan M. F., Alektiar K., Russo P. (2003). Adult spermatic cord sarcomas: management and results. *Annals of Surgical Oncology*.

[6] Fisher C., Goldblum J. R., Epstein J. I., Montgomery E. (2001). Leiomyosarcoma of the paratesticular region. *The American Journal of Surgical Pathology*.

[7] Khoubehi B., Mishra V., Ali M., Motiwala H., Karim O. (2002). Adult paratesticular tumours. *BJU International*.

[8] Coindre J. M., Terrier P., Bui N. B., Bonichon F., Collin F., Le Doussal V., Mandard A. M., Vilain M. O., Jacquemier J., Duplay H., Sastre X., Barlier C., Henry-Amar M., Macé-Lesech J., Contesso G. (1996). Prognostic factors in adult patients with locally controlled soft tissue sarcoma: a study of 546 patients from the French Federation of Cancer Centers Sarcoma Group. *Journal of Clinical Oncology*.

[9] Mondaini N., Palli D., Saieva C. (2005). Clinical characteristics and overall survival in genitourinary sarcomas treated with curative intent: a multicenter study. *European Urology*.

[10] Rodríguez D., Barrisford G. W., Sanchez A., Preston M. A., Kreydin E. I., Olumi A. F. (2014). Primary spermatic cord tumors: disease characteristics, prognostic factors, and treatment outcomes. *Urologic Oncology: Seminars and Original Investigations*.

[11] Stefano R., Anant D., James H. (2014). Prognostic factors and outcome of spermatic cord sarcoma. *Annals of Surgical Oncology*.

[12] Ballo M. T., Zagars G. K., Pisters P. W., Feig B. W., Patel S. R., Von Eschenbach A. C. (2001). Spermatic cord sarcoma: outcome, patterns of failure and management. *Journal of Urology*.

[13] Yuen V. T. H., Kirby S. D., Woo Y. C. (2011). Leiomyosarcoma of the epididymis: 2 cases and review of the literature. *Journal of the Canadian Urological Association*.

[14] Froehner M., Lossnitzer A., Manseck A., Koch R., Noack B., Wirth M. P. (2000). Favorable long-term outcome in adult genitourinary low-grade sarcoma. *Urology*.

[15] Galosi A. B., Scarpelli M., Mazzucchelli R. (2014). Adult primary paratesticular mesenchymal tumors with emphasis on a case presentation and discussion of spermatic cord leiomyosarcoma. *Diagnostic Pathology*.

